# A phase I, randomized, controlled, dose-ranging study of investigational acellular pertussis (aP) and reduced tetanus-diphtheria-acellular pertussis (TdaP) booster vaccines in adults

**DOI:** 10.1080/21645515.2017.1385686

**Published:** 2017-11-27

**Authors:** Geert Leroux-Roels, Maria Lattanzi, Claudia Dovali Solis, Mario Contorni, Marco Costantini, Luca Moraschini, Monia Bardelli, Sylvie Bertholet, Erica Borgogni, Francesca Buricchi, Rocco Cantisani, Elisa Faenzi, Oretta Finco, Rosanna Leuzzi, Mariagrazia Pizza, Domenico Rosa, Francesca Schiavetti, Anja Seubert, Fabiana Spensieri, Gianfranco Volpini, Luisanna Zedda, Giuseppe Del Giudice, Ilaria Galgani

**Affiliations:** a Centre for Vaccinology, Ghent University and University Hospital, Ghent, Belgium; b GSK, Vaccines, Siena, Italy

**Keywords:** adults, genetically detoxified PT, immunogenicity, persistence, pertussis, safety

## Abstract

Despite high vaccination coverage worldwide, pertussis has re-emerged in many countries. This randomized, controlled, observer-blind phase I study and extension study in Belgium (March 2012–June 2015) assessed safety and immunogenicity of investigational acellular pertussis vaccines containing genetically detoxified pertussis toxin (PT) (NCT01529645; NCT02382913).

420 healthy adults (average age: 26.8 ± 5.5 years, 60% female) were randomized to 1 of 10 vaccine groups: 3 investigational aP vaccines (containing pertussis antigens PT, filamentous hemagglutinin [FHA] and pertactin [PRN] at different dosages), 6 investigational TdaP (additionally containing tetanus toxoid [TT] and diphtheria toxoid [DT]), and 1 TdaP comparator containing chemically inactivated PT. Antibody responses were evaluated on days 1, 8, 30, 180, 365, and approximately 3 years post-booster vaccination. Cell-mediated immune responses and PT neutralization were evaluated in a subset of participants in pre-selected groups. Local and systemic adverse events (AEs), and unsolicited AEs were collected through day 7 and 30, respectively; serious AEs and AEs leading to study withdrawal were collected through day 365 post-vaccination.

Antibody responses against pertussis antigens peaked at day 30 post-vaccination and then declined but remained above baseline level at approximately 3 years post-vaccination. Responses to FHA and PRN were correlated to antigen dose. Antibody responses specific to PT, toxin neutralization activity and persistence induced by investigational formulations were similar or significantly higher than the licensed vaccine, despite lower PT doses. Of 15 serious AEs, none were considered vaccination-related; 1 led to study withdrawal (premature labor, day 364; aP4 group).

This study confirmed the potential benefits of genetically detoxified PT antigen. All investigational study formulations were well tolerated.

## Introduction

Pertussis, also known as whooping cough, is a highly contagious respiratory disease caused by the bacterium *Bordetella pertussis (B. pertussis)*. All age groups are susceptible to pertussis; however, the most severe symptoms occur in infants and young children, in whom potentially fatal complications such as convulsions, bronchopneumonia and encephalopathy may occur.^1–3^ Household exposure is considered to play an important role in the spread of the disease.^4–6^ A previous study demonstrated that 35% to 55% of infant cases could be prevented if immunity to pertussis in parents was maintained or boosted.

Despite the widespread vaccine availability and high vaccination coverage of primary and booster tetanus, diphtheria and acellular pertussis (aP) vaccinations, the incidence of pertussis continues to rise in many countries, with the highest morbidity and mortality rates in infants too young to be vaccinated.^7–10^ It was estimated in 2008 that 16 million cases of pertussis occurred worldwide, and 195,000 children died from the disease, an incidence that owes to insufficient coverage or compliance in pediatric immunization,^11–12^ as well as to resurgence in countries with high vaccination coverage: a high incidence of pertussis was observed in some developed countries (Australia, Portugal, United Kingdom, United States) that switched vaccination programs from vaccines that contained whole cell pertussis (wP) to aP.^13^ Health agencies in the United States and European Union recommend booster vaccine administration to adults in close contact with infants to reduce the risk of the disease.^14–15^ As a result of resurgence and increased incidence of infant mortality in 2010–2012, several countries including the United States and United Kingdom recommend maternal vaccination during pregnancy to protect newborns against pertussis.^14–16^

Currently available aP vaccines with pertussis toxin (PT) and other *B. pertussis* antigens have proven their safety and efficacy in large-scale clinical trials.^17–18^ However, the increased incidence of pertussis despite high aP coverage suggests that current vaccines induce immunity that may not be long-lasting against circulating strains. The limited longevity of protection against pertussis is also observed following natural infection.^19^ Therefore, a new generation of vaccines (primary and booster combinations) capable of inducing enhanced and long-lasting immunity is warranted. Such vaccines would also be useful for those countries currently using wP combinations, as neither vaccination nor natural immunity are able to confer life-long protection.

In order to improve disease control and induce enhanced immunity, an alternative method of detoxifying the PT was developed. The genetically detoxified PT (9K/129G) is an inactivated PT mutant that has been shown to be a superior immunogen as compared to the chemically detoxified PT that is currently used in licensed vaccines.^20–21^ The investigational aP booster vaccine comprising the genetically detoxified PT contains 2 other pertussis antigens (pertactin [PRN] and filamentous hemagglutinin [FHA]), and can be administered alone or in combination with tetanus toxoid (TT) and diphtheria toxoid (DT). The 3 pertussis components had already been included in a DTaP vaccine licensed for pediatric immunization in the 1990's (Triacelluvax, Chiron S.p.A.). Previous studies have shown that vaccine to be clinically efficacious in infants^22^ and to elicit long-lasting protection up through six years of life^23–24^ but it was withdrawn from the market in 2002 for commercial reasons.^25^

The phase I studies (main randomized clinical study and its extension study) presented here were conducted to assess the safety and antibody responses (with persistence up to 3 years) of different doses of investigational aP and tetanus-diphtheria-acellular pertussis (TdaP; adsorbed, reduced antigen content) vaccines, containing the genetically detoxified PT, as compared to a licensed TdaP vaccine (Boostrix, GSK) with chemically detoxified PT in healthy adults aged 18 through 40 years. Cell-mediated immunity (CMI) responses and PT neutralizing titers were evaluated in a subset of participants.

## Results

### Enrolment, study flow and demographics

**Figure 1.**
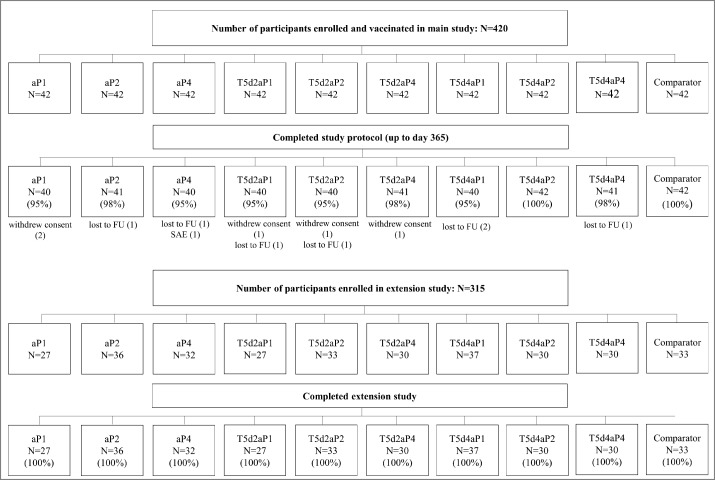
Flowchart main study and extension study. Footnote: FU, follow-up; N, number of participants in each group; SAE, serious adverse event.

A total of 420 participants (average age: 26.8 ± 5.5 years, 60% female) were enrolled and vaccinated in the main study. Of these participants, 407 (97%) completed the study protocol up to day 365 (Fig. 1). Reasons for premature study withdrawal were lost to follow-up (n = 7), withdrawal of consent (n = 5), and serious adverse event (SAE) (n = 1; participant from aP4 group withdrew herself after experiencing an SAE [premature labor] at day 364). Of the originally enrolled 420 participants in the main study, 315 participated in the extension study (range 27 to 37 of participants across groups). All participants completed the extension study (Fig. 1).

Participants enrolled in the main study were randomized into 10 equally-sized study groups of 42 participants each as outlined in Table 1. The baseline demographic characteristics of the enrolled participants in the main study and extension study are presented in Table 2. Vaccine groups were similar with respect to age, weight and height, and almost all study participants were of white heritage. Overall, a higher percentage of female than male participants was enrolled.

**Table 1.** Study groups and vaccine formulation.

	Day 1					Day 30
	Antigen dose					
	PT (μg)	FHA (μg)	PRN (μg)	DT (Lf)	TT (Lf)	
**aP Booster**						
aP1	1	1	2	0	0	Td-pur
aP2	2	2	4	0	0	Td-pur
aP4	4	4	8	0	0	Td-pur
**TdaP Booster**						
T5d2aP1^*^	1	1	2	2	5	Placebo
T5d2aP2	2	2	4	2	5	Placebo
T5d2aP4^*^	4	4	8	2	5	Placebo
T5d4aP1	1	1	2	4	5	Placebo
T5d4aP2	2	2	4	4	5	Placebo
T5d4aP4	4	4	8	4	5	Placebo
Comparator^*^	8	8	2.5	2.5	5	Placebo

* Selected groups for CMI analyses.

Licensed comparator is Boostrix (GSK). Td-pur: Licensed tetanus and diphtheria vaccine (Novartis Vaccines and Diagnostics)

aP: acellular pertussis; CMI: cell-mediated immunity; DT: diphtheria toxoid; FHA: filamentous hemagglutinin; Lf: limit of flocculation; PRN: pertactin; PT: pertussis toxin; TdaP: tetanus, diphtheria, acellular pertussis booster vaccine; TT: tetanus toxoid.

**Table 2.** Study population demographics.

Main study	aP1 N = 42	aP2 N = 42	aP4 N = 42	T5d2aP1 N = 42	T5d2aP2 N = 42	T5d2aP4 N = 42	T5d4aP1 N = 42	T5d4aP2 N = 42	T5d4aP4 N = 42	Comparator N = 42
Age, years	26.6 ± 5.6	26.8 ± 5.1	27.4 ± 6.5	26.8 ± 5.7	27.7 ± 5.9	25.8 ± 5.2	25.1 ± 4.2	27.5 ± 5.1	27.2 ± 5.6	27.4 ± 6.4
Female, n (%)	24 (57)	28 (67)	25 (60)	23 (55)	27 (64)	24 (57)	29 (69)	19 (45)	27 (64)	24 (57)
Male, n (%)	18 (43)	14 (33)	17 (40)	19 (45)	15 (36)	18 (43)	13 (31)	23 (55)	15 (36)	18 (43)
Ethnicity, n (%)										
Asian	0	0	0	0	1 (2)	0	0	0	0	0
Black	0	0	0	0	1 (2)	1 (2)	0	0	0	0
White	42 (100)	42 (100)	42 (100)	42 (100)	40 (95)	41 (98)	41 (98)	42 (100)	42 (100)	42 (100)
Other	0	0	0	0	0	0	1 (2)			
Weight, kg	68.4 ± 10.5	70.1 ± 9.9	69.0 ± 11.6	68.9 ± 12.8	65.4 ± 10.0	69.1 ± 11.4	67.7 ± 11.9	68.8 ± 13.5	69.7 ± 12.6	70.3 ± 13.5
Height, cm	173.9 ± 8.5	171.9 ± 8.3	172.3 ± 8.4	172.8 ± 7.8	172.3 ± 10.3	173.2 ± 8.2	169.9 ± 8.5	175.8 ± 8.6	171.8 ± 9.3	173.1 ± 7.9
Met entry criteria, n (%)	42 (100)	42 (100)	42 (100)	42 (100)	42 (100)	42 (100)	41 (98)	41 (98)	42 (100)	42 (100)
Extension Study	aP1 N = 27	aP2 N = 36	aP4 N = 32	T5d2aP1 N = 27	T5d2aP2 N = 33	T5d2aP4 N = 30	T5d4aP1 N = 37	T5d4aP2 N = 30	T5d4aP4 N = 30	Comparator N = 33
Age, years	30.2 ± 5.7	29.5 ± 4.9	30.3 ± 6.4	31.3 ± 6.3	30.9 ± 5.7	30.0 ± 5.6	28.2 ± 4.4	30.1 ± 4.6	31.3 ± 5.9	31.5 ± 6.7
Female, n (%)	17 (63)	25 (69)	19 (59)	11 (41)	19 (58)	16 (53)	27 (73)	13 (43)	19 (63)	21 (64)
Male, n (%)	10 (37)	11 (31)	13 (41)	16 (59)	14 (42)	14 (47)	10 (27)	17 (57)	11 (37)	12 (36)
Ethnicity, n (%)										
Asian	0	0	0	0	1 (3)	0	0	0	0	0
Black	0	0	0	0	1 (3)	1 (3)	0	0	0	0
White	27 (100)	36 (100)	32 (100)	27 (100)	31 (94)	29 (97)	36 (97)	30 (100)	30 (100)	33 (100)
Other	0	0	0	0	0	0	1 (3)			
Weight, kg	67.2 ± 8.4	71.1 ± 10.7	69.1 ± 11.5	72.4 ± 12.6	67.7 ± 10.7	69.9 ± 12.2	68.1 ± 12.2	70.6 ± 15.1	74.0 ± 11.7	72.5 ± 15.8
Height, cm	172.6 ± 9.0	171.1 ± 8.6	171.2 ± 8.5	174.2 ± 7.9	173.2 ± 10.7	172.9 ± 8.0	169.0 ± 8.0	175.8 ± 8.9	172.7 ± 9.6	172.1 ± 8.2
Met entry criteria, n (%)	27 (100)	36 (100)	32 (100)	27 (100)	33 (100)	30 (100)	37 (100)	30 (100)	30 (100)	33 (100)

Age, weight and height are expressed as mean ± standard deviation. Licensed comparator is Boostrix (GSK).

aP: acellular pertussis; N: number of participants in each group; n %: number (percentage).

### Safety and reactogenicity

All 420 enrolled participants were exposed to the study vaccines and 418 participants contributed to the safety analyses; 2 participants (1 in group aP4 and 1 in group T5d4aP1) were excluded for not providing any post-baseline safety data. Overall, the safety profile of the investigational aP and TdaP formulations was comparable to the licensed TdaP booster vaccine.

At least one reactogenicity sign post-vaccination was reported by similar numbers of participants in the aP groups (81% to 88%) and in the licensed comparator group (86%); in the TdaP group, at least one reactogenicity sign post-vaccination was reported by 93% to 98% participants. Similarly, solicited local adverse events (AEs) were reported by 71% to 79% of participants in the aP groups, 83% to 98% in the TdaP groups and by 79% in the comparator group. Solicited systemic AEs were reported by similar numbers of participants in all groups, i.e., 48% to 64% of participants in the aP groups, 43% to 66% in the TdaP groups and 50% in the licensed comparator group. No relevant trends towards higher incidences of solicited AEs with increasing antigen doses were observed in the investigational vaccine groups.

The most commonly reported local AE was injection site pain, experienced by 71% to 98% of participants in each group (Table 3). Severe injection site pain was reported by maximum 5% of participants across groups. Erythema was reported by 0% to 14% participants, induration by 2% to 22%; and pruritus by 0% to 14% across groups, with no reports of severe reactions. The most commonly reported systemic AEs occurring across all vaccine groups were fatigue (29% to 49%) and headache (19% to 40%) (Table 3). Severe fatigue was reported by maximum 7% of participants per group, severe headache by maximum 5%. Nausea was experienced by 2% to 17%, myalgia by 10% to 29%, and arthralgia by 0% to 17% of participants across groups, with only few severe reports (up to 5% across groups). Fever ≥38.0°C was rare, ranging from 0% to 7% across groups. There were no participants with fever ≥40C. Therapeutic use of analgesics and antipyretics was reported in 5% to 20% of participants across vaccine groups (Table 3).

**Table 3.** Number (percentage) of participants experiencing solicited local* and systemic adverse events and other indicators of reactogenicity within 7 days of vaccination.

	aP1 N = 42	aP2 N = 42	aP4 N = 41	T5d2aP1 N = 42	T5d2aP2 N = 42	T5d2aP4 N = 42	T5d4aP1 N = 41	T5d4aP2 N = 42	T5d4aP4 N = 42	Comparator N = 42
Erythema*	0	1 (2)	1 (2)	1 (2)	2 (5)	6 (14)	3 (7)	2 (5)	4 (10)	3 (7)
Induration*	3 (7)	3 (7)	1 (2)	6 (14)	1 (2)	7 (17)	9 (22)	4 (10)	4 (10)	6 (14)
Pain*	30 (71)	32 (76)	31 (76)	35 (83)	38 (90)	36 (86)	40 (98)	35 (83)	39 (93)	31 (74)
Severe	0	1 (2)	0	0	1 (2)	1 (2)	0	2 (5)	1 (2)	0
Pruritus*	0	4 (10)	1 (2)	3 (7)	2 (5)	5 (12)	3 (7)	3 (7)	6 (14)	5 (12)
Nausea	3 (7)	6 (14)	2 (5)	1 (2)	2 (5)	5 (12)	4 (10)	6 (14)	7 (17)	1 (2)
Severe	1 (2)	0	1 (2)	0	0	0	1 (2)	1 (2)	0	0
Myalgia	10 (24)	8 (19)	4 (10)	8 (19)	10 (24)	11 (26)	12 (29)	8 (19)	9 (21)	11 (26)
Severe	1 (2)	1 (2)	0	1 (2)	0	0	0	0	2 (5)	0
Arthralgia	2 (5)	0	3 (7)	4 (10)	1 (2)	3 (7)	4 (10)	4 (10)	7 (17)	1 (2)
Severe	1 (2)	0	0	0	0	0	0	0	0	0
Headache	17 (40)	13 (31)	12 (29)	8 (19)	13 (31)	13 (31)	16 (39)	14 (33)	17 (40)	13 (31)
Severe	1 (2)	0	0	2 (5)	1 (2)	2 (5)	2 (5)	2 (5)	2 (5)	1 (2)
Fatigue	19 (45)	14 (33)	13 (32)	13 (31)	15 (36)	18 (43)	20 (49)	15 (36)	16 (38)	12 (29)
Severe	3 (7)	2 (5)	0	2 (5)	0	1 (2)	2 (5)	0	2 (5)	0
Body temperature										
≥ 38C	1 (2)	0	1 (2)	2 (5)	0	0	3 (7)	2 (5)	0	2 (5)
≥ 40C	0	0	0	0	0	0	0	0	0	0
Use of analgesics and antipyretics										
Prophylactic	0	1 (2)	0	0	1 (2)	1 (2)	2 (5)	1 (2)	2 (5)	1 (2)
Therapeutic	6 (14)	7 (17)	2 (5)	6 (14)	8 (19)	3 (7)	8 (20)	7 (17)	7 (17)	8 (19)

Erythema and induration with diameter > 100 mm were classified as severe. No cases of severe erythema, induration, or pruritus were reported. Values represent number of reported cases (percentage). Licensed comparator is Boostrix (GSK).

aP: acellular pertussis; N: number of participants in each group.

The overall mean numeric rating scale (NRS) scores^26^ in the groups were very low (range 0.68 to 1.51 at 60 minutes post-vaccination, 0.84 to 1.77 at 6 hours post-vaccination, on a 0 to 10 scale), with slightly higher mean values in the TdaP groups (0.87–1.51 at 1 hour and 1.10–1.77 at 6 hours post-vaccination in the TdaP groups, compared to 0.80–0.99 and 0.84–0.99 in the aP groups; Table S1) For the responses to the likelihood of vaccination, the majority of participants (67% to 90% at day 1, 55% to 88% at day 8) across vaccine groups ‘strongly agreed’ that they were likely to undergo repeated vaccination after being administered the study vaccination (Table S1).

Unsolicited AEs occurred in 29% to 67% (17% to 31% considered at least possibly related) of participants in the aP groups, 31% to 50% (10% to 26% considered at least possibly related) in the TdaP groups and 43% (24% considered at least possibly related) in the licensed comparator group. The most frequently reported unsolicited AEs (by preferred term) were upper respiratory tract infection (up to 12%) and headache (up to 10%) for aP groups, upper respiratory tract infection, oropharyngeal pain, and headache (all up to 10%) for TdaP groups, and headache and injection site induration (both up to 5%) for the comparator group. Across groups, the most common at least possibly related AEs were injection site movement impairment, injection site pain, and fatigue (all up to 7%).

A total of 15 SAEs were reported by 14 participants, none of which were considered related to the study vaccination (Table S2) No deaths occurred in this study.

### Antigen-specific antibody responses

Analyses of antibody responses were performed on the per-protocol (PP) data sets, which included 383 (91%) of the total of 420 participants at days 30, 372 (89%) at day 180 and 383 (91%) at day 365, and 300/315 (95%) of participants approximately 3 years after vaccine administration (on day 1 of the extension study) (Table S3).

### Antibody response against pertussis antigens (PT, PRN, FHA)

**Figure 2.**
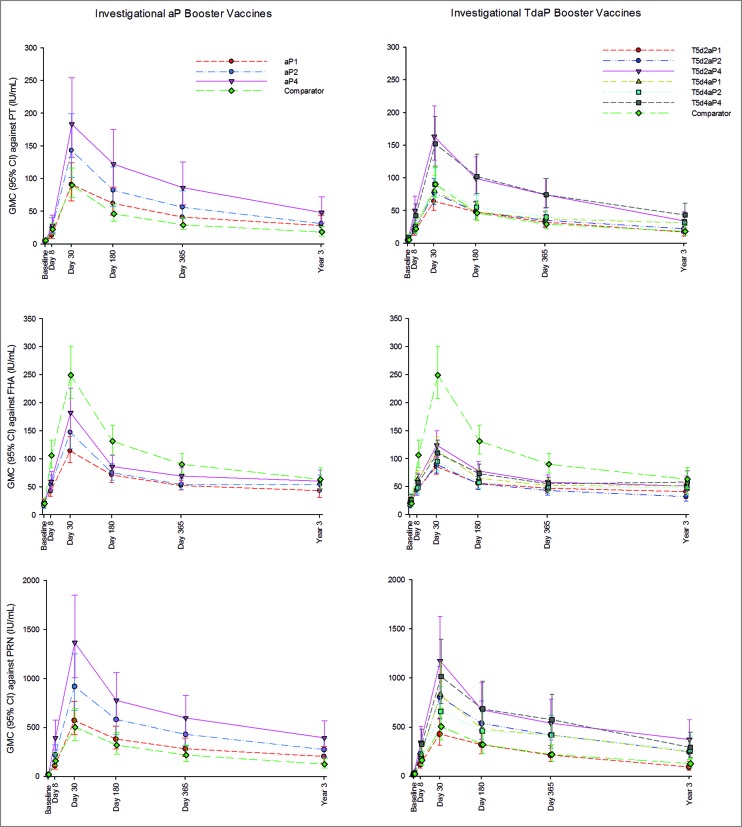
Geometric mean concentrations and 95% confidence intervals against pertussis antigens PT, FHA and PRN in investigational aP and TdaP booster groups and licensed comparator group from day 1 through year 3 post-vaccination. Footnote: aP, acellular pertussis; CI, confidence interval; FHA, filamentous hemagglutinin; GMC, geometric mean concentration; IU/mL, International Units per milliliter; PRN, pertactin; PT, pertussis toxin; TdaP, tetanus diphtheria acellular pertussis.

Geometric mean concentrations (GMCs) against pertussis antigens PT, FHA and PRN from baseline up to 3 years post-vaccination are presented in Fig. 2. Baseline values were comparable across groups. Overall, antibody responses against pertussis antigens peaked at the day 30 timepoint after the booster dose and then waned over the following 3 years, but were still above baseline levels.

For PT, the highest antibody responses were observed in the investigational formulations containing 4 µg genetically detoxified PT (groups aP4, T5d2aP4, and T5d4aP4) at all timepoints, including day 365 and 3 years post-vaccination. Even formulations with 2 µg of genetically detoxified PT induced antibody responses similar to or higher than the licensed comparator containing 8 µg of chemically inactivated PT (Fig. 2).

A statistically significant increase in antibody responses at day 30 post-vaccination (evidenced by the ratio of GMCs and their 95% confidence intervals [CIs]) against PT was observed in aP2, aP4, T5d2aP4 and T5d4aP4 groups, as compared to the licensed vaccine. Day 30 GMCs across groups ranged from 62 to 182, being lowest in the T5d2aP1 group and highest in the aP4 group. Percentages of participants with ≥2-fold increase in levels of antibodies against PT were 94% to 100% (aP formulations), 89% to 100% (TdaP formulations) and 100% (licensed comparator); ≥4-fold increase was observed in 92% to 100% (aP formulations), 76% to 100% (TdaP formulations) and 98% (licensed comparator) participants.

For all vaccine groups, antibody levels against PT at 3 years post-vaccination were higher than at baseline (7.21 to 11-fold higher GMCs in the aP groups, 3.4 to 6.67-fold higher GMCs in the TdaP groups; 4.28-fold higher in the comparator group). Participants who received vaccine formulations containing 4 µg PT demonstrated statistically significant higher persistence of anti-PT antibody responses at 3 years post-vaccination than participants who received the licensed comparator containing the double dose of PT. For aP and TdaP formulations containing 1or 2 µg PT, persistence of antibody against PT was comparable or statistically higher (T5d4aP1 and T5d4aP2 groups, respectively) than for the licensed TdaP vaccine (Table 4).

**Table 4.** Ratio of GMCs with 95% confidence intervals of aP and TdaP booster doses relative to licensed comparator against pertussis PT, FHA and PRN antigens approximately 3 years post-vaccination (day 1 extension study).

	aP1: Comparator	aP2: Comparator	aP4: Comparator	T5d2aP1: Comparator	T5d2aP2: Comparator	T5d2aP4: Comparator	T5d4aP1: Comparator	T5d4aP2: Comparator	T5d4aP4: Comparator
PT	1.54 (0.85-2.81)	1.70 (0.97-2.98)	2.65 (1.50-4.70)	0.92 (0.56-1.52)	1.20 (0.74-1.94)	1.86 (1.13-3.05)	1.71 (1.07-2.75)	1.69 (1.03-2.77)	2.37 (1.45-3.87)
FHA	0.68 (0.44-1.04)	0.86 (0.57-1.28)	0.95 (0.63-1.43)	0.64 (0.42-0.99)	0.52 (0.34-0.78)	0.81 (0.53-1.23)	0.80 (0.54-1.21)	0.76 (0.50-1.17)	0.92 (0.60-1.39)
PRN	1.64 (0.95-2.82)	2.19 (1.31-3.65)	3.14 (1.87-5.28)	0.74 (0.40-1.36)	1.97 (1.11-3.51)	2.96 (1.63-5.36)	1.96 (1.11-3.46)	2.11 (1.17-3.82)	2.32 (1.29-4.18)

When the 95% confidence interval does not contain 1, there is an indication of a statistically significant difference. Licensed comparator is Boostrix (GSK).

aP: acellular pertussis; GMC: geometric mean concentration; FHA: filamentous hemagglutinin; PRN: pertactin; PT: pertussis toxin; TdaP: tetanus diphtheria acellular pertussis.

The investigational aP and TdaP groups demonstrated lower antibody responses against the FHA antigen at all timepoints than the licensed TdaP vaccine (Fig. 2). The aP and TdaP formulations contained 1/8 to 1/2 of the dose of FHA as compared to the licensed vaccine.

In all aP and TdaP study groups, antibody responses against FHA at day 30 post-vaccination were statistically significant lower than those elicited by the licensed vaccine. GMCs at day 30 following booster vaccinations ranged from 83 (T5d2aP1) to 241 (licensed TdaP vaccine) international units (IU)/mL.

Percentages of participants with ≥2-fold increase in the levels of antibodies against FHA were 85% to 94% for the aP groups, 76% to 95% for the TdaP groups and 95% for the licensed vaccine; ≥4-fold increase was observed in 59% to 83% (aP groups), 46% to 61% (TdaP groups) and 83% (licensed vaccine) participants.

Antibody persistence 3 years post-vaccination against FHA was higher than at baseline for all vaccine groups (2.25 to 3.25-fold rise across the aP groups, 1.67 to 2.19-fold rise across the TdaP groups; 3.36-fold rise comparator). The level of persisting anti-FHA antibodies 3 years post-vaccination was lower in the aP and TdaP groups than in the licensed comparator group, with significant differences observed for T5d2aP1 and T5d2aP2 groups (Table 4).

For PRN, antibody responses were 0.32–2.71-fold higher in aP4, 2.10–2.96-fold higher in T5d2aP4, and 1.98–2.60-fold higher in the T5d4aP4 study groups (PRN content 8 μg) at all timepoints, as compared with the licensed comparator (PRN content 2.5 μg) (Fig. 2).

At 30 days after administration of the booster vaccine, significant increases in antibody responses against PRN were noted for aP2, aP4, T5d2aP2, T5d2aP4, T5d4aP1 and T5d4aP4 groups, as compared to the licensed vaccine. Post-vaccination day 30 GMCs ranged from 445 to 1384, with the lowest observed response in the T5d2aP1 group, and the highest observed response in the aP4 group. Percentages of participants with ≥2-fold increase in PRN antigen were 100% (all aP groups), 92% to 100% (TdaP groups) and 100% (licensed comparator); percentages of participants with ≥4-fold increase were 92% to 100% (aP groups), 70% to 98% (TdaP groups) and 90% (licensed comparator).

At 3 years following vaccination, anti-PRN GMCs for all groups were higher than at baseline as indicated by an 11 to 20-fold (aP groups), 7.65 to 13-fold (TdaP group) and 6.61-fold (comparator) rise in year 3 GMCs relative to day 1. A higher persistence of antibody responses at 3 years post-vaccination was observed for aP and TdaP groups as compared to the licensed vaccine, with significant group differences for all investigational formulations except aP1 and T5d2aP1 (Table 4).

### Antibody response against tetanus and diphtheria (TT and DT)

On day 30 post-vaccination with TdaP or licensed comparator, participants had antibody concentrations above the seroprotection level (i.e., ≥0.1 IU/mL) against TT (97% to 100%) and DT antigens (95% to 100%). The 2 limit of flocculation (Lf) diphtheria antigen dose was sufficient to achieve seroprotection levels against diphtheria in 95% to 98% of participants. Percentages of participants with antibodies above cut-off values ≥1.0 IU/mL were 97% to 100% (investigational TdaP) and 98% (licensed comparator) against the TT antigen and 68% to 89% (investigational TdaP, highest in the groups with the 4Lf diphtheria antigen dose) and 78% (licensed comparator) against the DT antigen.

**Figure 3.**
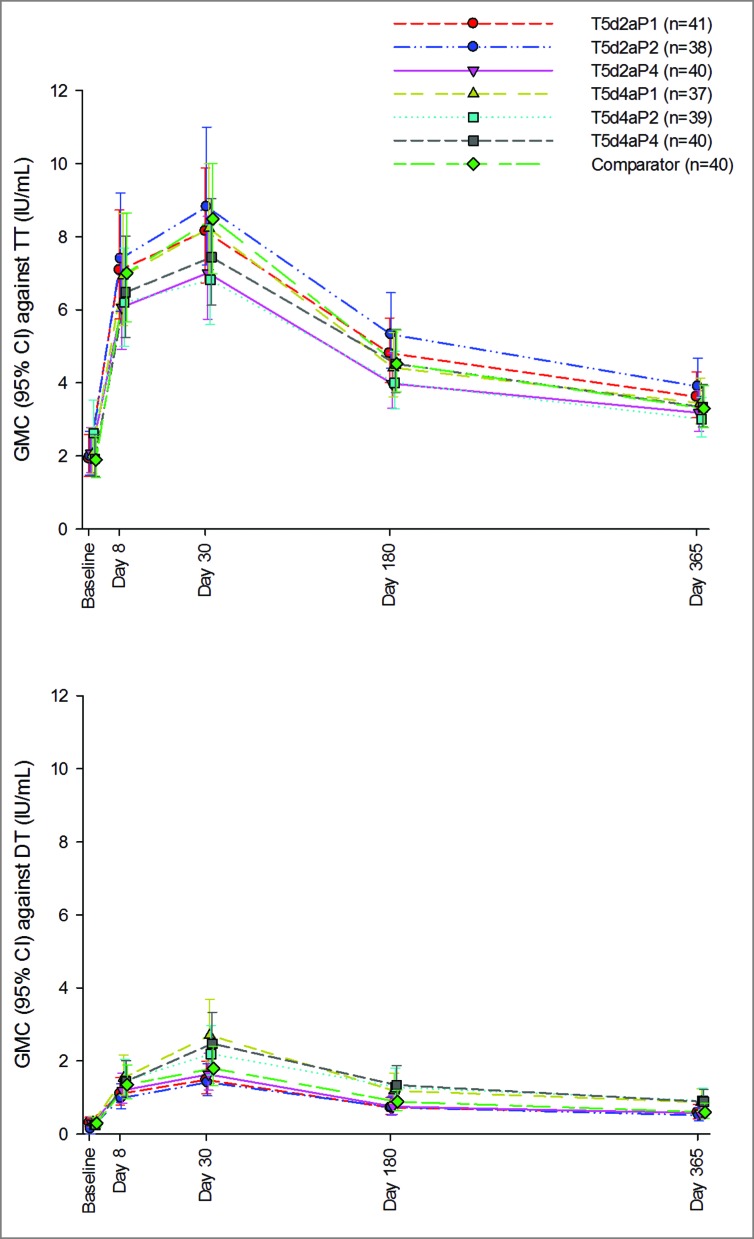
Geometric mean concentrations and 95% confidence intervals against tetanus and diphtheria antigens in investigational TdaP booster groups and licensed comparator group from day 1 up through day 365 post-vaccination. Footnote: CI, confidence interval; DT, diphtheria toxoid; GMC, geometric mean concentration; IU/mL, International Units per milliliter; n, maximum number of participants with available results, TT, tetanus toxoid; TdaP, tetanus diphtheria acellular pertussis.

GMCs against TT and DT from baseline up to 1 year post-vaccination are presented in Fig. 3. Baseline GMCs were similar in the TdaP and comparator groups. Values had increased on day 30 in all groups, with comparable GMCs for TdaP and comparator groups against the TT antigen. GMCs against the DT antigen were highest with the TdaP formulations with the 4Lf DT antigen dose. Antibody levels waned in all groups at day 180 and day 365, but remained above baseline levels across all groups.

### PT neutralization

PT neutralization analysis was performed post-hoc in the same subsets of participants from the same three groups selected for CMI analyses.

**Figure 4.**
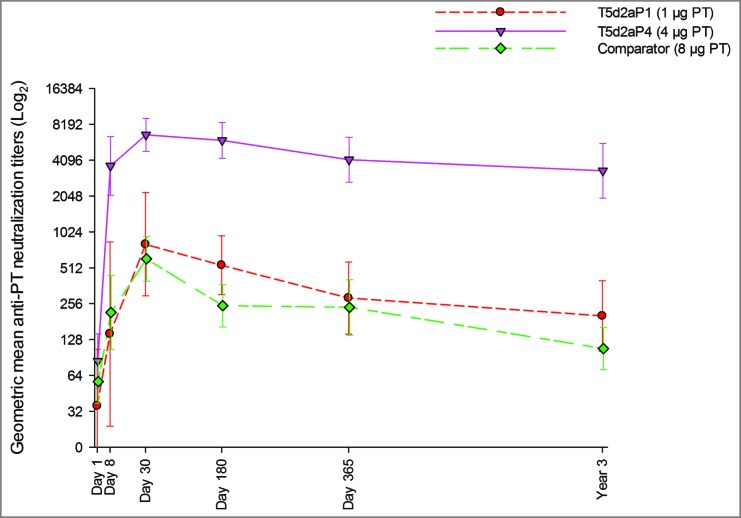
Geometric mean anti-pertussis neutralizing titers and 95% confidence intervals from day 1 through year 3 post-vaccination. Footnote: PT, pertussis toxin.

PT neutralization analysis was performed to assess the functionality of anti-PT antibodies induced by the formulations with 1 and 4 μg PT in comparison to the licensed comparator containing 8 μg PT. Geometric mean titers at all timepoints are shown in Fig. 4. Analysis of neutralizing titers revealed that formulation with 4 μg resulted in a markedly higher anti-PT antibody functionality at all timepoints. High titers were observed already at day 8, with a following peak at day 30; titers then showed a slight decline at later timepoints high functional activity still persisted over 3 years. The 1 μg formulation and the licensed comparator lead to a similar path of neutralization titers: an increase of GMT was observed starting from day 8 with a peak at day 30; titers then waned over the following 3 years but still remained higher than the baseline values.

### Antigen-specific T- and B-cell responses (CMI)

**Figure 5.**
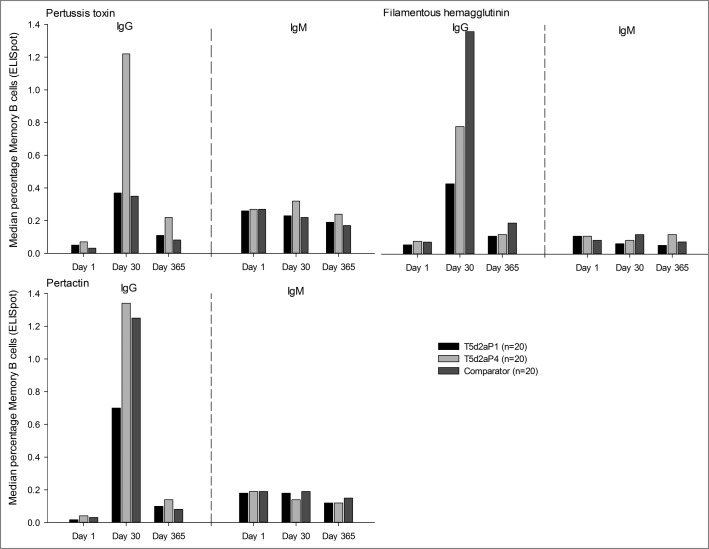
Median percentages of Memory B Cells (ELISpot) against pertussis antigens PT, FHA and PRN in high and low dose aP dose TdaP groups and licensed comparator from day 1 through day 365 post-vaccination. Footnote: aP, acellular pertussis; ELISpot, enzyme-linked immunospot assay; FHA, filamentous hemagglutinin; IgG, immunoglobulin G; IgM, immunoglobulin M; n, number of participants with available results; PRN, pertactin; PT, pertussis toxin; TdaP, tetanus diphtheria acellular pertussis.

Antigen-specific T- and B-cell responses were assessed in a subset of 20 participants from each of the T5d2aP1, T5d2aP4, and licensed comparator groups. Irrespective of timepoint and class of antibody (IgG or IgM), over 65% of participants across the 3 vaccine groups presented quantifiable frequencies of memory B cells (MBC) for each vaccine antigen. Higher frequencies of IgG-positive MBC were observed on day 30 relative to day 1, whereas no increased frequencies were observed for IgM-positive MBC. MBC frequencies decreased from day 30 to day 365, but remained generally still higher than those observed on day 1. A higher frequency of IgG-positive PT-specific MBC was observed in the high aP dose TdaP group (T5d2aP4) at all timepoints as compared with the lower dose groups and licensed comparator. Frequency of IgG-positive FHA-specific MBC at day 30 post-vaccination was higher in the licensed TdaP vaccine, with the lowest observed responses in the investigational TdaP formulation containing the lower aP dose. There was no significant difference in PRN responses between the different groups (Fig. 5).

On day 8 post-vaccination, at least 85% of participants presented quantifiable frequencies (values greater than 0) of plasmablasts (PB) secreting IgG antibodies against each vaccine-related antigen. Although there were no major differences across the 3 vaccine groups in percentage of participants with measurable frequencies of IgG-secreting PB, there was a significantly higher frequency of PT-specific PB in the high aP dose Tdap group than in the low-dose group (p < 0.0015). Comparable frequencies of PB were observed in the high aP dose Tdap group and licensed comparator group against all vaccine antigens except FHA antigen; the IgG-secreting PB against FHA antigen were higher in the comparator group than in the T5d2aP1 group (p < 0.0004) and T5d2aP4 group (p < 0.0004). A low percentage (0% to 5%) of participants had IgM-secreting PB against DT and PT, while up to 35% of participants produced antibodies against FHA and PRN (data not shown).

**Figure 6.**
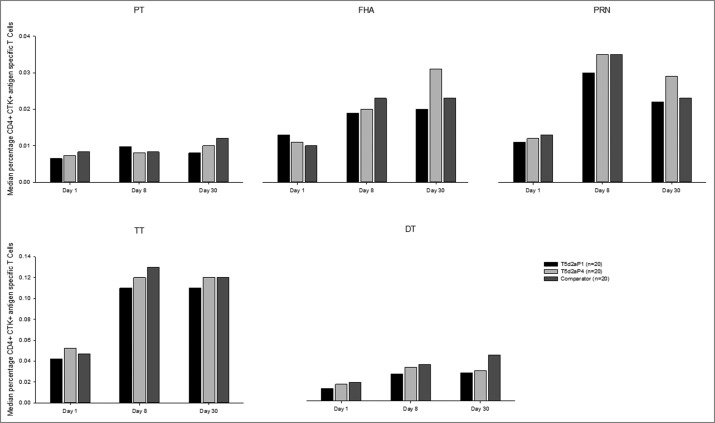
Median percentages of antigen-specific CD4^+^ T cells in high and low aP dose groups and licensed comparator at baseline and days 8 and 30 post-vaccination. Footnote: CTK, cytokine; DT, diphtheria toxoid; FHA, filamentous hemagglutinin; n, maximum number of participants with available results; PRN, pertactin; PT, pertussis toxin; TdaP, tetanus, diphtheria, acellular pertussis; TT, tetanus toxoid.

All participants (100%) had quantifiable frequencies of antigen-specific CD4^+^ T cells for all antigens at all analyzed timepoints (day 1, day 8 and day 30). The frequencies of CD4^+^ T cells against vaccine antigens were consistently higher at days 8 and 30 than on day 1. However, the T cell-specific responses against PT antigen were very weak (Fig. 6). Analyses of the functional profile of antigen-specific CD4+ T cells demonstrated that on day 1, the majority of the participants had detectable antigen-specific CD4+ T cells expressing at least one of the following cytokines: interferon gamma (IFN-γ), interleukin-2 (IL-2), IL-17, IL-21, and tumor necrosis factor alpha (TNF-α), excluding IL-13. On day 8 and day 30, the frequency of cytokine-positive CD4+ T cells increased in all vaccine groups and against all tested antigens except PT. In general, fewer or no IL-13-producing T cells were observed at all timepoints analyzed, demonstrating that the response induced by vaccination was principally Th-1-like (data not shown).

## Discussion

The present study evaluated the safety and antibody responses of different doses of investigational aP and TdaP vaccines, containing a genetically detoxified PT, as compared to a licensed TdaP vaccine containing a chemically detoxified PT, in a population of healthy adults.

The results demonstrate that all investigational study formulations were well tolerated, with reactogenicity and safety profiles similar to those observed for the licensed comparator. Adverse reactions were transient, mainly mild to moderate in severity, and there was no evidence of increasing rates of events with increasing dose. The overall mean NRS scores were very low, and comparable across groups. On the NRS scale of 0–10 that was used, a value of 0 is no pain while 1–3 is mild pain.^26^ In chronic pain studies, a reduction of approximately two points or a reduction of approximately 30% in the pain intensity NRS has been reported to represent a clinically important difference.^27^

The most frequently reported local reaction to either vaccine formulation was pain at the injection site; the most commonly reported systemic reactions were fatigue and headache. Rates of fever ≥38°C were rare, with no cases of severe fever ≥40C. There was no vaccine-related SAE throughout the study period and none of the participants withdrew prematurely due to a non-serious AE; 1 participant from the aP4 group withdrew herself due to an SAE (premature labor on day 364). These findings are in line with the known safety profile of the licensed TdaP vaccines.^28–29^

All vaccine formulations induced an antibody response against the tested antigens. Antibody responses against *B. pertussis* peaked at day 30 after the booster vaccination and then waned in the following 3 years post-vaccination, but remained above baseline levels. The genetically detoxified PT dose of 4 μg/mL induced a statistically significantly higher antibody response and persistence of antibodies to the PT antigen as compared to the licensed vaccine containing the double PT dose; even 1/4^th^ of the dose of genetically detoxified PT induced antibody responses similar to or higher than those observed for the licensed comparator. Of importance, the higher performance of genetically detoxified PT was markedly evident at functional level, giving a stronger neutralizing activity both at the level of early response and at antibody persistence level. While chemical detoxification is effective, it also greatly alters the immunological properties of the toxin through its impact on the structure of the toxin; in contrast, genetically detoxified PT maintains all functional and immunological properties, making it a superior antigen compared to chemically detoxified PT.^20^ The improved PT-specific antibody responses, despite lower antigen doses, are consistent with previous efficacy trials in infants showing that the genetically inactivated PT induced a stronger antibody response than the chemically detoxified toxin.^22–24^ These studies also demonstrated long-lasting protection through the first six years of life.^23–24^ Superiority of the genetically detoxified PT antigen was further supported by the significantly enhanced numbers of PT-specific IgG MBC cells, when compared to the licensed comparator.

Antibody responses to FHA and PRN antigens reflected the quantity of vaccine per dose. For PRN, antibody responses and persistence of antibodies were especially higher with the investigational formulations containing 8 μg PRN dose (aP4, T5d2aP4, and T5d4aP4 groups) as compared with the licensed comparator containing 2.5 μg PRN dose. FHA doses in all investigational formulations were lower than in the licensed vaccine and were not sufficient to induce an immune response higher than or similar to that obtained with the licensed comparator, indicating the need to increase the FHA content.

No established serological correlates of protection for pertussis have been identified, hampering estimation of the protective potential after pertussis booster vaccination.^30^ However, for the aP part of the vaccine, results can be compared with the efficacy trial of the licensed TdaP comparator in adults and adolescents, demonstrating that on the basis of primary pertussis case definition, vaccine protection was 92%.^31^ Also, previous studies reported persistence of antibodies above pre-booster values against all vaccine antigens up to 3 years after vaccination with the licensed TdaP comparator in adults and 10 years after vaccination in young adults,^32–33^ and pertussis-specific antibody and CMI levels above the pre-booster levels measured 5 years earlier.^34^

Analyses of B cell responses were in line with the findings of antibody responses. Increased frequencies of antigen-specific CD4^+^ T cells against vaccine antigens were detected on days 8 and 30 relative to baseline. The fast increase of the frequency of these cells supports the presence of a sizeable pool of memory antigen-specific CD4^+^ T cells which could be quickly expanded following vaccination. The results reported in this study do not allow discriminating whether the genetically detoxified and the chemically detoxified PT induced qualitatively different CD4^+^ T cell populations. This may be due to the low number of participants tested in each group and to the intrinsic variability of the results. Another explanation, although not mutually exclusive with the first, could be the fact that all participants were primed during their pediatric age with a wP vaccine, which is known to drive the immune response towards a Th1-type functional phenotype, as compared to aP vaccines,^35^ and probably to provide an “imprinting” of the response that would have not been changed upon boosting with the vaccines used in the study.

IgG-positive MBC were highest on day 30 after the booster vaccination and then waned up to day 365, but remained above pre-booster levels. Frequencies of PB, MBC and CD4^+^ T cells in the high aP dose group were similar to or higher than those observed for the licensed comparator, which strengthens the arguments that the most appropriate dosage for aP is 4 μg for PT, 4 μg for FHA, and 8 μg for PRN. The high frequency of MBC and PB committed to produce antigen-specific IgG more than IgM speaks in favor of a persisting immunological memory originally primed by vaccination at the pediatric age, despite the lack of detectable antibodies before vaccination. The strong response induced with the genetically detoxified PT also suggests that the genetically detoxified toxin was able to boost a response after priming with cellular wP vaccine. The low CD4+ T cell response observed after in vitro stimulation with the PT, despite the strong antibody and B-cell response, may be due to the treatment PT underwent in order to avoid its intrinsic ability to stimulate T cells non-specifically.

Study limitations include the absence of predefined hypotheses to be tested formally, despite the relatively large number of participants included for a phase I study. Moreover, no adjustment for multiple testing was performed, so some spurious statistically significant differences between groups may have been observed by chance. For the analysis of antigen-specific cellular responses, the small number of participants analyzed (20 for each group) and the frequently observed non-detectable values for cytokine expression suggested performing just a descriptive analysis, and made it difficult to draw generalizable conclusions. Additionally, the vaccination history of the participants has not been registered, but DTwP vaccination was introduced in Belgium in 1961–1962, and considering the age of participants at study enrolment, most — if not all — participants should have received in infancy the recommended scheme of three doses of DTwP at the ages of 3, 4 and 5 months. Acellular pertussis vaccination was introduced in Belgium in 2001, and since participants in our study were 18–40 years old, we could infer that none of the participants have received acellular pertussis vaccine in infancy. Finally, no comparisons were performed between responses from a primary schedule with wP-containing vaccine versus aP-containing vaccine, which would have allowed for further conclusions in terms of global value.

**Figure 7.**
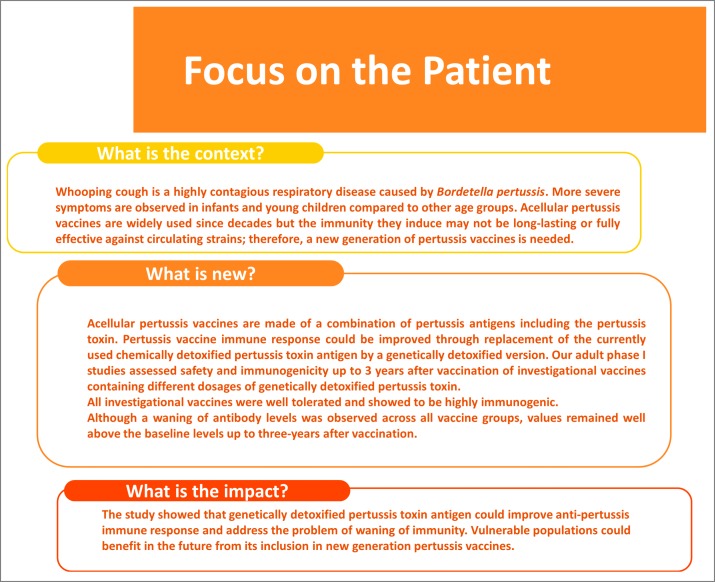
Focus on Patient Section.

A lay language graphical summary contextualizing the results and potential clinical research relevance and impact is displayed in the Focus on Patient Section (Fig. 7).

Altogether, this study demonstrates that all study vaccines were well tolerated and confirmed the potential benefit of the genetically detoxified PT antigen. Next steps in development may include further dose finding, assessment of vaccine safety and immunogenicity in vulnerable populations such as elderly and pregnant women, and evaluation of the potential for co-administrations with other vaccines. Also, potential inclusion of genetically detoxified PT in primary childhood vaccines — similar to Triacelluvax (DTaP) that demonstrated a long lasting clinical protection^22–24^ or in more complex combination vaccines — could be considered. Boostability of the genetically detoxified PT formulation itself also remains to be explored. Further studies on adult booster vaccination will be required to assess impact of this vaccine on adult disease, on the transmission dynamics of pertussis in the whole population, and consequently on the protection it may provide for infants who are too young to be immunized.

## Patients and methods

### Study design and objectives

The current phase I randomized, controlled, observer-blind, dose-ranging study and extension study was conducted at 1 site in Belgium (Centre for Vaccinology, Ghent University Hospital) between March 2012 and June 2015 (Clinicaltrials.gov identifiers: main study: NCT01529645; extension study: NCT02382913). The study was undertaken according to Good Clinical Practice and the Declaration of Helsinki. The ethic review committee of the participating center approved the study protocol, and written informed consent was obtained from every participant prior to enrolment.

The safety objectives included assessment of the safety profiles of 3 aP booster vaccines with different antigen doses and 6 different Tdap booster vaccines to the licensed TdaP vaccine in terms of solicited local and systemic AEs and patient reported outcomes for the period of 7 days after vaccination; and unsolicited AEs for the period of 30 days after vaccination.

The immunogenicity objective was to select 1or 2 study vaccines from each of the aP and TdaP investigational formulations by comparison of antibody responses with the licensed TdaP vaccine and to each other at 30 days after vaccination, provided that safety profiles were comparable. The exploratory objectives included persistence of antibody responses against each antigenic component at days 180 and 365 post-vaccination and persistence of anti-PT, anti-FHA and anti-PRN antibody levels approximately 3 years after vaccine administration (day 1 of the extension study); immune persistence as measured by CMI responses in subsets of participants from selected groups (Table 1). at day 1, day 30, and day 365 after vaccination; comparison of early onset of immune response as measured by CMI and antibody response in subsets of participants from selected groups (Table 1) to the licensed TdaP vaccine and to each other at day 8; evaluation of the correlation between NRS and the solicited local reaction of injection site pain in participants receiving study vaccination.

PT neutralization activity was evaluated by a retrospective laboratory research at days 1, 8, 30 and 365 and approximately 3 years after vaccine administration (day 1 of the extension study) in the same subsets of participants selected for antigen-specific T and B cell analysis.

### Study participants

A total of 420 healthy adults were randomized to 1 of 10 study groups: 3 investigational aP (groups: aP1, aP2, and aP4), 6 investigational TdaP (groups: T5d2aP1, T5d2aP2, T5d2aP4, T5d4aP1, T5d4aP2, T5d4aP4) and 1 licensed TdaP comparator (Table 1). Randomization was performed according to a validated web-based system (Biostatistics and Clinical Data Management department, Novartis). An equal number of participants (42) were included in each group. Subsets of 20 participants in each of the pre-selected groups — T5d2aP1 (low aP dose TdaP), T5d2aP4 (high aP dose TdaP) and licensed TdaP comparator groups — were included in the CMI analyses.

Eligible study participants for the main study were healthy adults of either sex between 18 and 40 years of age at the time of enrolment. Participants were excluded if they had received any vaccines against tetanus, diphtheria or pertussis (aP or wP), if they had been diagnosed with pertussis disease or if they had a household exposure with pertussis within the past 8 years*.* Other exclusion criteria were contraindications to the licensed TdaP comparator or Td-pur (Novartis Vaccines and Diagnostics) as specified within the summary of product characteristics; a significant infection or oral body temperature ≥38°C within 3 days of the intended date of vaccination; known reactions to vaccine components; any progressive or severe neurologic disease, seizure disorder or Guillain-Barré syndrome; behavioral or cognitive impairment that might interfere with the person's ability to participate in the study; any medical history or (serious) illness likely to interfere with the results; known or suspected immune disease or impairment including the administration of steroids; abnormalities of splenic or thymic function; known bleeding diathesis or any condition associated with a prolonged bleeding time; a body mass index greater than 35kg/m^2^; previous receipt of any other vaccine within 14 days (inactivated vaccines) or 28 days (live vaccines) or intent to receive any other vaccines within 28 days from the study vaccines; participation or intent to participate in any clinical trial 30 days prior to study start or during the time of enrolment; substance or alcohol abuse within the past 2 years; family members of study staff and pregnant or breast-feeding women. Women of childbearing potential had to be committed to using birth control measures for the duration of the study, and they had to have used birth control measures for at least 2 months before study participation. Eligible study participants for the extension study included adults who had been previously enrolled and had completed the main study and who had received the appropriate booster vaccine according to the randomization group.

### Vaccination

Study vaccines were prepared as a 0.5mL white suspension in a prefilled, glass syringe for a single intramuscular (IM) administration. The investigational vaccine formulations as listed in Table 1 included the following combinations of pertussis antigens (1, 2 or 4 μg PT; 1, 2 or 4 μg FHA and 2, 4 or 8 μg PRN), DT (0, 2 or 4 Lf) and TT (0 or 5 Lf). The licensed comparator TdaP vaccine (Boostrix) included the following active ingredients per dose: pertussis antigens (8 μg PT; 8 μg FHA; 2.5 μg PRN), DT (2.5 Lf) and TT (5 Lf). Boostrix is indicated for active booster immunization against TdaP as a single dose in individuals 10 years and older. All study participants received a single 0.5mL IM vaccine dose on day 1 in the deltoid region of the upper non-dominant arm. Designated unblinded study personnel, who otherwise did not participate in the evaluation of the participants during the trial, administered the vaccine formulations. To ensure Td booster vaccination in all groups, participants who received aP alone on day 1 received the licensed vaccine Td-pur on day 30, all other participants in the remaining groups who received TdaP on day 1 received placebo (saline) on day 30.

### Safety analyses

Study participants were provided with diary cards and the frequency and severity of a predefined set of solicited local and systemic AEs and other reactogenicity indicators were recorded on a daily basis from day 1 to day 7 following vaccination. After vaccination, all participants were observed for at least 30 minutes at the study site to monitor for immediate AEs. Solicited local AEs included erythema, induration, pain and pruritus. Solicited systemic AEs included nausea, myalgia, arthralgia, headache and fatigue. Other indicators of reactogenicity were oral body temperature (≥38C) and the use of analgesics/antipyretics. Additional outcomes included the NRS to measure the participant's perception of pain at the injection site ranging from 0 (no pain) to 10 (worst imaginable pain) and the Likert scale to evaluate the participant's perception on ‘the likelihood to undergo repeat vaccination after receipt of study vaccination’ (1 strongly disagree, 5 strongly agree). All AEs were collected from day 1 through day 30. SAEs and AEs leading to study withdrawal were recorded from day 1 through day 365. The severity of unsolicited AEs was categorized as mild, moderate, or severe, if they resulted in no limitation, some limitation, or inability to perform normal daily activities, respectively. Assessments of the causal relationship of unsolicited AEs to the vaccination were classified by the investigator as not related, possibly related, or probably related.

### Immunogenicity analyses

Blood samples were obtained for immunogenicity analyses at baseline (day 1, pre-vaccination), on days 8, 30, 180 and 365 post-vaccination and approximately 3 years after vaccine administration (i.e., on day 1 of the extension study). Sera were tested using validated methods at PPD, Inc., Vaccines & Biologics (Wayne, PA United States). Peripheral blood mononuclear cells (PBMC) were analyzed at the Novartis Vaccine and Diagnostics, Translational Medicine Laboratory, Siena, Italy.

Antibody responses to PT, FHA and PRN were assessed by standard enzyme-linked immunosorbent assay (ELISA) and expressed as GMC, and percentages of participants with at least 2-fold and 4-fold antibody concentration increase from pre- to post-vaccination. Anti-diphtheria and anti-tetanus antibodies were determined using ELISA with protective levels set at ≥0.1 IU/mL.^36–37^ Results were expressed as percentages of participants with antibody levels ≥0.1 IU/mL, 1.0 IU/mL and GMCs. For tetanus, seroprotection was defined as antibody levels >0.1 IU/mL, while antibody levels >1.0 IU/mL were considered indicative of long term protection.^38^ To evaluate the persistence of pertussis antibody levels, geometric mean ratios (GMRs) of the post-vaccination to pre-vaccination concentrations were calculated as PT, FHA and PRN concentrations on day 1 of the extension study approximately 3 years after vaccination, relative to days 1, 8, 30, 180 and 365.

Frequency and functional profile of CD4^+^ T cells specific for vaccine antigens were assessed by polychromatic flow cytometry as described in the literature.^39^ Antigen-specific CD3+ CD4+ T lymphocytes were analyzed by measuring the frequency of T cells that produced the following cytokines in response to the *in vitro* stimulation: IL-2, IL-13, IL-17, IL-21, IFN-γ and TNF-α. A subset of participants of the groups T5d2aP1 (low AP dose TdaP), T5d2aP4 (high AP dose TdaP) and licensed comparator were analyzed (20 participants/group) at days 1, 8 and 30. In addition, the B lymphocyte response was evaluated by assessing the frequencies of antigen-specific MBC at days 1, 30 and 365 and PB at day 8 through enzyme-linked immunospot assay (ELISpot).^40^

PT neutralizing titers were measured as described in the literature.^41^ Two-fold serially diluted sera were pre-incubated with active PT and added to Chinese hamster ovary-K1 cells, followed by evaluation of morphological alterations (clustered phenotype) by light microscopy. Endpoint titers are the reciprocal of the highest dilution able to inhibit cell clustering.

### Statistical analyses

Statistical analyses were performed using Statistical Analyses System (SAS) software version 9.1 (SAS Institute, Cary, NC, United States). A minimal sample of 40 evaluable participants per vaccine group was estimated to provide sufficient power to examine the primary study objective. As this was an exploratory study, no formal statistical hypothesis was tested.

The primary immunogenicity objective was to select 1 or 2 study vaccines (out of 3 aP booster vaccines and out of 6 TdaP booster vaccines) by comparison of antibody responses with the licensed TdaP vaccine as well as to each other; comparisons were based on antibody responses to each antigenic component at 30 days after vaccination. Two dose groups were considered statistically different if the 2-sided 95% CI around the difference of their means of the log_10_ transformed data did not contain the value 0 for at least 1 antigen and, similarly, if the 2-sided 95% CI around the difference of group proportions did not include the value 0 for at least 1 antigen. GMCs, GMRs, percentages of participants with 2- or 4- fold changes for the aP antigens (PT, FHA, PRN) and percentages of participants with anti-diphtheria and anti-tetanus antibodies above cut-off values ≥0.1 IU/mL and ≥1.0 IU/mL were calculated.

The estimation of GMTs for the PT neutralization activity at each timepoint and for each vaccine group was done using ANOVA models with fixed factor for regimen group. CIs were calculated using the same models.

Safety data was summarized for each vaccine group, providing the frequency and proportion of participants reporting an event. Immunogenicity analyses were run on the PP set, which consisted of participants who received the relevant dose of vaccine correctly, provided at least one evaluable serum sample at the relevant timepoints, and had no major protocol violations (Supplementary. Table 3) A major deviation was defined as a protocol deviation that was considered to have a significant impact on the immunogenicity result of the participant. Safety was analyzed for all participants who provided post-vaccination data.

Boostrix is a trade mark of the GSK group of companies. Td-pur is a trade mark of Novartis Vaccines and Diagnostics.

## Supplementary Material

KHVI_A_1385686_Supplemental.docx

## Abbreviations

AE: adverse event
aP: acellular pertussis
*B. pertussis*: *Bordetella pertussis*
CI: confidence interval
CMI: cell-mediated immunity
DT: diphtheria toxoid
DTaP: diphtheria, tetanus, acellular pertussis primary vaccine
ELISA: enzyme-linked immunosorbent assay
ELISpot: enzyme-linked immunospot assay
FHA: filamentous hemagglutinin
GMC: geometric mean concentration
GMR: geometric mean ratio
IM: intramuscular
IL-2: interleukin-2
IL-13: interleukin-13
IL-17: interleukin-17
IL-21: interleukin-21
IFN-γ: interferon gamma
IU: international unit
Lf: limit of flocculation
MBC: memory B cells
NRS: numeric rating scale
PB: plasmablasts
PBMC: Peripheral blood mononuclear cells
PP: per-protocol
PRN: pertactin
PT: pertussis toxin
SAE: serious adverse event
SAS: Statistical Analysis System
TdaP: tetanus, diphtheria, acellular pertussis booster vaccine
TT: tetanus toxoid
TNF-α: tumor necrosis factor alpha
wP: whole-cell pertussis

## References

[1] Greenberg DP, von Konig CH, Heininger U Health burden of pertussis in infants and children. Pediatr Infect Dis J. 2005;24:S39–43; doi:10.1097/01.inf.0000160911.65632.e1. PMID:15876922 15876922

[2] Centers for Disease Control and Prevention. Pertussis (Whooping Cough). Complications; [accessed 1 August 2016]. http://www.cdc.gov/pertussis/about/complications.html

[3] Tanaka M, Vitek CR, Pascual FB, Bisgard KM, Tate JE, Murphy TV Trends in pertussis among infants in the United States, 1980–1999. JAMA. 2003;290:2968–75. doi:10.1001/jama.290.22.2968. PMID:14665658 14665658

[4] Bisgard KM, Pascual FB, Ehresmann KR, Miller CA, Cianfrini C, Jennings CE, Rebmann CA, Gabel J, Schauer SL, Lett SM Infant pertussis: who was the source? Pediatr Infect Dis J. 2004;23:985–9. doi:10.1097/01.inf.0000145263.37198.2b. PMID:15545851 15545851

[5] de Greeff SC, Mooi FR, Westerhof A, Verbakel JM, Peeters MF, Heuvelman CJ, Notermans DW, Elvers LH, Schellekens JF, de Melker HE Pertussis disease burden in the household: How to protect young infants. Clin Infect Dis. 2010;50:1339–45. doi:10.1086/652281. PMID:20370464 20370464

[6] Wirsing von König CH, Postels-Multan S, Schmitt HJ, Bock HL Pertussis in adults: Ffrequency of transmission after household exposure. Lancet. 1995;346:1326–9. doi:10.1016/S0140-6736(95)92343-8. PMID:7475771 7475771

[7] Cortese MM, Baughman AL, Zhang R, Srivastava PU, Wallace GS Pertussis hospitalizations among infants in the United States, 1993 to 2004. Pediatrics. 2008;121:484–92. doi:10.1542/peds.2007-1393. PMID:18310196 18310196

[8] European Centre For Disease Prevention and Control. Surveillance Report. Annual epidemiological report vaccine-preventable diseases 2014 [accessed 1 August 2016]. http://ecdc.europa.eu/en/publications/Publications/AER-2014-VPD-FINAL.pdf

[9] Centers for Disease Control and Prevention. Pertussis (Whooping Cough). Surveillance and Reporting. [accessed 30 May 2017]. http://www.cdc.gov/pertussis/surv-reporting.html

[10] Clark TA. Changing pertussis epidemiology: Everything old is new again. J Infect Dis. 2014;209:978–81. doi:10.1093/infdis/jiu001. PMID:24626532 24626532

[11] Black RE, Cousens S, Johnson HL, Lawn JE, Rudan I, Bassani DG, Jha P, Campbell H, Walker CF, Cibulskis R, et al. Global, regional, and national causes of child mortality in 2008: A systematic analysis. Lancet. 2010;375:1969–87. doi:10.1016/S0140-6736(10)60549-1. PMID:20466419 20466419

[12] Organization World Health Immunization, Vaccines and Biologicals – Data, statistics and graphics. [accessed 24 May 2017]. http://www.who.int/immunization/monitoring_surveillance/data/en/

[13] World Health Organization Pertussis vaccines: WHO position paper – August 2015. Weekly epidemiological record. 2015;35:433–60.

[14] Centers for Disease Control Prevention Updated recommendations for use of tetanus toxoid, reduced diphtheria toxoid and acellular pertussis vaccine (Tdap) in pregnant women and persons who have or anticipate having close contact with an infant aged <12 months. MMWR Morb Mortal Wkly Rep. 2011;60:1424–6. PMID:22012116 22012116

[15] Chiappini E, Stival A, Galli L, de Martino M Pertussis re-emergence in the post-vaccination era. BMC Infect Dis. 2013;13:151. doi:10.1186/1471-2334-13-151. PMID:23530907 23530907 PMC3623740

[16] Centers for Disease Control Prevention Updated recommendations for use of tetanus toxoid, reduced diphtheria toxoid, and acellular pertussis vaccine (Tdap) in pregnant women–Advisory Committee on Immunization Practices (ACIP), 2012. MMWR Morb Mortal Wkly Rep. 2013;62:131–5; PMID:23425962 23425962 PMC4604886

[17] McGirr A, Fisman DN Duration of pertussis immunity after DTaP immunization: a meta-analysis. Pediatrics. 2015;135:331–43. doi:10.1542/peds.2014-1729. PMID:25560446 25560446

[18] Zhang L, Prietsch SO, Axelsson I, Halperin SA Acellular vaccines for preventing whooping cough in children. Cochrane Database Syst Rev. 2014;9:CD001478. doi:10.1002/14651858.CD001478.pub6. 25228233 PMC9722541

[19] Wendelboe AM, Van Rie A, Salmaso S, Englund JA Duration of immunity against pertussis after natural infection or vaccination. Pediatr Infect Dis J. 2005;24:S58–61. doi:10.1097/01.inf.0000160914.59160.41. PMID:15876927 15876927

[20] Seubert A, D'Oro U, Scarselli M, Pizza M Genetically detoxified pertussis toxin (PT-9 K/129G): Implications for immunization and vaccines. Expert Rev Vaccines. 2014;13:1191–204. doi:10.1586/14760584.2014.942641. PMID:25183193 25183193

[21] Edwards KM, Meade BD, Decker MD, Reed GF, Rennels MB, Steinhoff MC, Anderson EL, Englund JA, Pichichero ME, Deloria MA Comparison of 13 acellular pertussis vaccines: Overview and serologic response. Pediatrics 1995;96:548–57. PMID:7659475 7659475

[22] Greco D, Salmaso S, Mastrantonio P, Giuliano M, Tozzi AE, Anemona A, Ciofi degli Atti ML, Giammanco A, Panei P, Blackwelder WC, et al. A controlled trial of two acellular vaccines and one whole-cell vaccine against pertussis. Progetto Pertosse Working Group. N Engl J Med. 1996;334:341–8. doi:10.1056/NEJM199602083340601. PMID:8538704 8538704

[23] Salmaso S, Mastrantonio P, Tozzi AE, Stefanelli P, Anemona A, Ciofi degli Atti ML, Giammanco A, Stage III WG Sustained efficacy during the first 6 years of life of 3-component acellular pertussis vaccines administered in infancy: the Italian experience. Pediatrics. 2001;108:E81. doi:10.1542/peds.108.5.e81. PMID:11694665 11694665

[24] Salmaso S, Mastrantonio P, Wassilak SG, Giuliano M, Anemona A, Giammanco A, Tozzi AE, Ciofi degli Atti ML, Greco D Persistence of protection through 33 months of age provided by immunization in infancy with two three-component acellular pertussis vaccines. Stage II Working Group. Vaccine. 1998;16:1270–5. doi:10.1016/S0264-410X(98)00040-1. PMID:9682390 9682390

[25] EMEA The European Agency for the Evaluation of Medicinal Products. Public Statement on Triacelluvax. Withdrawal of the marketing authorisation in the European Union. [accessed March 13, 2017].

[26] The Numeric Pain Rating Scale Instructions [accessed 24 May 2017]. http://www.rehabmeasures.org/PDF%20Library/Numeric%20Pain%20Rating%20Scale%20Instructions.pdf

[27] Farrar JT, Young JP Jr, LaMoreaux L, Werth JL, Poole RM Clinical importance of changes in chronic pain intensity measured on an 11-point numerical pain rating scale. Pain. 2001;94:149–58. doi:10.1016/S0304-3959(01)00349-9. PMID:11690728 11690728

[28] Li WC, Wu TZ, Huang YC, Huang LM Boostrix: a reduced-dose acellular pertussis vaccine for use in adolescents and adults. Expert Rev Vaccines. 2009;8:1317–27. doi:10.1586/erv.09.96. PMID:19803753 19803753

[29] Pichichero ME, Blatter MM, Kennedy WA, Hedrick J, Descamps D, Friedland LR Acellular pertussis vaccine booster combined with diphtheria and tetanus toxoids for adolescents. Pediatrics. 2006;117:1084–93. doi:10.1542/peds.2005-1759. PMID:16585302 16585302

[30] Plotkin SA. Complex correlates of protection after vaccination. Clin Infect Dis. 2013;56:1458–65. doi:10.1093/cid/cit048. PMID:23386629 23386629

[31] Ward JI, Cherry JD, Chang SJ, Partridge S, Lee H, Treanor J, Greenberg DP, Keitel W, Barenkamp S, Bernstein DI, et al. Efficacy of an acellular pertussis vaccine among adolescents and adults. N Engl J Med. 2005;353:1555–63. doi:10.1056/NEJMoa050824. PMID:16221778 16221778

[32] Vandermeulen C, Theeten H, Rathi N, Kuriyakose S, Han HH, Sokal E, Hoppenbrouwers K, Van Damme P Decennial administration in young adults of a reduced-antigen content diphtheria, tetanus, acellular pertussis vaccine containing two different concentrations of aluminium. Vaccine. 2015;33:3026–34. doi:10.1016/j.vaccine.2014.10.049. PMID:25613716 25613716

[33] Weston W, Messier M, Friedland LR, Wu X, Howe B Persistence of antibodies 3 years after booster vaccination of adults with combined acellular pertussis, diphtheria and tetanus toxoids vaccine. Vaccine. 2011;29:8483–6. doi:10.1016/j.vaccine.2011.09.063. PMID:21945698 21945698

[34] Edelman K, He Q, Makinen J, Sahlberg A, Haanpera M, Schuerman L, Wolter J, Mertsola J Immunity to pertussis 5 years after booster immunization during adolescence. Clin Infect Dis. 2007;44:1271–7. doi:10.1086/514338. PMID:17443462 17443462

[35] Ausiello CM, Urbani F, la Sala A, Lande R, Cassone A Vaccine- and antigen-dependent type 1 and type 2 cytokine induction after primary vaccination of infants with whole-cell or acellular pertussis vaccines. Infect Immun. 1997;65:2168–74. PMID:9169747 9169747 10.1128/iai.65.6.2168-2174.1997 PMC175299

[36] von Hunolstein C Aggerbeck H, Andrews N, Berbers G, Fievet-Groyne F, Maple PA, Olander RM, Raux M, Tischer A European sero-epidemiology network: Standardisation of the results of diphtheria antitoxin assays. Vaccine. 2000;18:3287–96. doi:10.1016/S0264-410X(00)00125-0. PMID:10869774 10869774

[37] World Health Organization (WHO) The immunological basis for immunization series. Module 3: Tetanus update. 2006; [accessed 3 August 2010]. http://apps.who.int/iris/bitstream/10665/43687/1/9789241595551_eng.pdf

[38] Maple PA, Jones CS, Wall EC, Vyseb A, Edmunds WJ, Andrews NJ, Miller E Immunity to diphtheria and tetanus in England and Wales. Vaccine. 2000;19:167–73. doi:10.1016/S0264-410X(00)00184-5. PMID:10930669 10930669

[39] Galli G, Medini D, Borgogni E, Zedda L, Bardelli M, Malzone C, Nuti S, Tavarini S, Sammicheli C, Hilbert AK, et al. Adjuvanted H5N1 vaccine induces early CD4+ T cell response that predicts long-term persistence of protective antibody levels. Proc Natl Acad Sci U S A. 2009;106:3877–82. doi:10.1073/pnas.0813390106. PMID:19237568 19237568 PMC2646626

[40] Jahnmatz M, Kesa G, Netterlid E, Buisman AM, Thorstensson R, Ahlborg N Optimization of a human IgG B-cell ELISpot assay for the analysis of vaccine-induced B-cell responses. J Immunol Methods. 2013;391:50–9. doi:10.1016/j.jim.2013.02.009. PMID:23454005 23454005

[41] Hewlett EL, Sauer KT, Myers GA, Cowell JL, Guerrant RL Induction of a novel morphological response in Chinese hamster ovary cells by pertussis toxin. Infect Immun. 1983;40:1198–203. PMID:6682833 6682833 10.1128/iai.40.3.1198-1203.1983 PMC348177

