# Supplementary material for: A phase I, randomized, controlled, dose-ranging study of investigational acellular pertussis (aP) and reduced tetanus-diphtheria-acellular pertussis (TdaP) booster vaccines in adults
Source: Hum Vaccin Immunother. 2017 Nov 27;14(1):45–58. doi: 10.1080/21645515.2017.1385686 (PMC5791588; doi:10.1080/21645515.2017.1385686)
Supplement: KHVI_A_1385686_Supplemental.docx [file khvi-14-01-1385686-s001.docx]

**Supplementary tables**

**Supplementary table 1: Numeric rating scale scores and likelihood of repeat vaccination**

| **Main study** | **aP1**  **N=42** | **aP2**  **N=42** | **aP4**  **N=42** | **T5d2aP1**  **N=42** | **T5d2aP2**  **N=42** | **T5d2aP4**  **N=42** | **T5d4aP1**  **N=42** | **T5d4aP2**  **N=42** | **T5d4aP4**  **N=42** | **Comparator**  **N=42** |
| --- | --- | --- | --- | --- | --- | --- | --- | --- | --- | --- |
| **Mean numeric rating scale values:** | | | | | | | | | | |
| 60 min post-vac | 0.99±1.14 | 0.95±1.53 | 0.80±1.13 | 0.87±0.87 | 1.02±1.17 | 1.40±1.75 | 1.51±1.64 | 1.04±1.35 | 1.17±1.17 | 0.68±0.85 |
| 6 hours post-vac | 0.99±1.10 | 0.94±1.44 | 0.84±0.99 | 1.10±1.23 | 1.29±1.28 | 1.60±1.74 | 1.72±1.64 | 1.67±1.89 | 1.77±1.64 | 0.04±1.08 |
| **Likelihood of repeat vaccination: n (%)** | | | | | | | | | | |
| **Day 1:** |  |  |  |  |  |  |  |  |  |  |
| Strongly agree | 34 (81%) | 37 (88%) | 31 (74%) | 35 (83%) | 34 (81%) | 30 (71%) | 28 (67%) | 30 (71%) | 28 (67%) | 38 (90%) |
| Agree | 8 (19%) | 5 (12%) | 10 (24%) | 7 (17%) | 8 (19%) | 11 (26%) | 13 (31%) | 12 (29%) | 13 (31%) | 4 (10%) |
| Neither agree nor disagree | 0 | 0 | 1 (2%) | 0 | 0 | 1 (2%) | 1 (2%) | 0 | 1 (2%) | 0 |
| **Day 8:** |  |  |  |  |  |  |  |  |  |  |
| Strongly agree | 27 (64%) | 33 (79%) | 30 (71%) | 30 (71%) | 31 (74%) | 23 (55%) | 24 (57%) | 25 (60%) | 27 (64%) | 37 (88%) |
| Agree | 13 (31%) | 6 (14%) | 10 (24%) | 11 (26%) | 11 (26%) | 17 (40%) | 13 (31%) | 17 (40%) | 13 (31%) | 4 (10%) |
| Neither agree nor disagree | 1 (2%) | 0 | 1 (2%) | 1 (2%) | 0 | 1 (2%) | 3 (7%) | 0 | 2 (5%) | 0 |
| Disagree | 0 | 1 (2%) | 0 | 0 | 0 | 1 (2%) | 1 (2%) | 0 | 0 | 0 |
| Strongly disagree | 1 (2%) | 1 (2%) | 0 | 0 | 0 | 0 | 0 | 0 | 0 | 1 (2%) |
| Not available | 0 | 1 | 1 | 0 | 0 | 0 | 1 | 0 | 0 | 0 |

N, number of participants in each group; n, number of participants with available results.

**Supplementary table 2. Serious adverse events reported during the study**

| Group | Preferred Term | Onset  (Study day) | Duration (days) | Outcome | Relationship to Study Vaccine |
| --- | --- | --- | --- | --- | --- |
| **aP1** |  |  |  |  |  |
|  | Ankle fracture | 85 | Continued | Not Recovered | None |
|  | Depression | - | Continued | Not Recovered | None |
|  | Dysthymic disorder | - | 104 | Recovered | None |
|  | Foot deformity | - | Continued | Not Recovered | None |
|  | Peritonsillar abscess | 346 | 13 | Recovered | None |
| **aP2** |  |  |  |  |  |
|  | Polymyalgia rheumatica | - | Continued | Not Recovered | None |
| **aP4** |  |  |  |  |  |
|  | Premature labor | 364 | 9 | Recovered | None |
| **T5d2aP1** |  |  |  |  |  |
|  | Gastroenteritis | 157 | 34 | Recovered | None |
| **T5d2aP4** |  |  |  |  |  |
|  | Appendicitis | 23 | 7 | Recovered | None |
| **T5d4aP1** |  |  |  |  |  |
|  | Chronic sinusitis | 5 | 357 | Recovered | None |
| **T5d4aP2** |  |  |  |  |  |
|  | Crohn's disease | 25 | 72 | Recovered with sequelae | None |
|  | Pneumothorax | 259 | 23-53 | Recovered | None |
| **T5d4aP4** |  |  |  |  |  |
|  | Psychotic disorder | 131 | 61 | Recovered | None |
|  | Pericarditis | 223 | 41 | Recovered | None |
| **Comparator** |  |  |  |  |  |
|  | Tonsillitis streptococcal | 134 | 16 | Recovered | None |

**Supplementary table 3: Per-protocol data sets and reasons for exclusion**

1. **Day 30**

| **Analysis Set** | **aP1** | **aP2** | **aP4** | **T5d2aP1** | **T5d2aP2** | **T5d2aP4** | **T5d4aP1** | **T5d4aP2** | **T5d4aP4** | **Comparator** | **Total** |
| --- | --- | --- | --- | --- | --- | --- | --- | --- | --- | --- | --- |
| Enrolled Set (N) | 42 | 42 | 42 | 42 | 42 | 42 | 42 | 42 | 42 | 42 | 420 |
| Exposed Set | 42 | 42 | 42 | 42 | 42 | 42 | 42 | 42 | 42 | 42 | 420 |
| FAS | 42 | 42 | 42 | 42 | 42 | 42 | 42 | 42 | 42 | 42 | 420 |
| Blood draw out of window at Day 30 | 3 | 3 | 2 | - | - | 2 | - | - | - | - | 10 |
| No blood draw at Day 30 | - | - | 1 | - | - | - | - | - | - | - | 1 |
| Did not meet entry criteria | - | - | - | - | - | - | 1 | 1 | - | - | 2 |
| Received a wrong vaccine | - | 3 | 2 | - | 2 | 2 | 3 | 2 | 2 | 2 | 18 |
| Received an excluded concomitant medication | - | - | - | - | - | - | - | 1 | - | - | 1 |
| Withdrew from the study | - | 1 | 1 | 1 | 2 | - | 1 | - | - | - | 6 |
| Participants eligible for PPS Day 30 | 39 | 35 | 36 | 41 | 38 | 38 | 37 | 39 | 40 | 40 | 383 |

1. **Day 180**

| **Analysis Set** | **aP1** | **aP2** | **aP4** | **T5d2aP1** | **T5d2aP2** | **T5d2aP4** | **T5d4aP1** | **T5d4aP2** | **T5d4aP4** | **Comparator** | **Total** |
| --- | --- | --- | --- | --- | --- | --- | --- | --- | --- | --- | --- |
| FAS (N) | 42 | 42 | 42 | 42 | 42 | 42 | 42 | 42 | 42 | 42 | 420 |
| Blood draw out of window at Day 180 | 2 | - | 1 | - | 1 | - | 1 | 3 | 1 | 1 | 10 |
| No blood draw at Day 180 | 1 | 2 | 3 | 1 | 1 | 2 | - | 1 | 2 | - | 13 |
| Did not meet entry criteria | - | - | - | - | - | - | 1 | 1 | - | - | 2 |
| Received a wrong vaccine | - | 3 | 2 | 0 | 2 | 2 | 3 | 2 | 2 | 2 | 18 |
| Received an excluded concomitant medication | - | - | - | - | - | - | - | 1 | - | - | 1 |
| Withdrew from the study | 1 | 1 | 2 | 1 | 2 | - | 2 | - | 1 | - | 10 |
| Participants eligible for PPS Day 180 | 39 | 36 | 36 | 40 | 36 | 38 | 35 | 36 | 37 | 39 | 372 |

1. **Day 365**

| **Analysis Set** | **aP1** | **aP2** | **aP4** | **T5d2aP1** | **T5d2aP2** | **T5d2aP4** | **T5d4aP1** | **T5d4aP2** | **T5d4aP4** | **Comparator** | **Total** |
| --- | --- | --- | --- | --- | --- | --- | --- | --- | --- | --- | --- |
| FAS (N) | 42 | 42 | 42 | 42 | 42 | 42 | 42 | 42 | 42 | 42 | 420 |
| Blood draw out of window at Day 365 | - | - | - | - | 2 | - | - | 1 | - | 1 | 4 |
| Did not meet entry criteria | - | - | - | - | - | - | 1 | 1 | - | - | 2 |
| Received a wrong vaccine | - | 3 | 2 | - | 2 | 2 | 3 | 2 | 2 | 2 | 18 |
| Received an excluded concomitant medication | - | - | - | - | - | - | - | 1 | - | - | 1 |
| Withdrew from the study | 2 | 1 | 2 | 2 | 2 | 1 | 2 | - | 1 | - | 13 |
| Participants eligible for PPS Day 365 | 40 | 38 | 38 | 40 | 37 | 39 | 36 | 38 | 39 | 40 | 383 |

1. **Year 3**

| **Analysis Set** | **aP1** | **aP2** | **aP4** | **T5d2aP1** | **T5d2aP2** | **T5d2aP4** | **T5d4aP1** | **T5d4aP2** | **T5d4aP4** | **Comparator** | **Total** |
| --- | --- | --- | --- | --- | --- | --- | --- | --- | --- | --- | --- |
| FAS (N) | 27 | 36 | 32 | 27 | 33 | 30 | 37 | 30 | 30 | 33 | 315 |
| Vaccination not according to the protocol | - | 2 | - | 1 | 2 | 2 | 4 | 2 | 1 | 1 | 15 |
| Participants eligible for PPS Year 3 | 27 | 34 | 32 | 26 | 31 | 28 | 33 | 28 | 29 | 32 | 300 |

N, number of participants in each group; FAS, full analysis set; PPS, per protocol set
